# Bio-Inspired Ionic Sensors: Transforming Natural Mechanisms into Sensory Technologies

**DOI:** 10.1007/s40820-025-01692-6

**Published:** 2025-03-12

**Authors:** Kyongtae Choi, Gibeom Lee, Min-Gyu Lee, Hee Jae Hwang, Kibeom Lee, Younghoon Lee

**Affiliations:** 1 Department of Mechanical Engineering, Kyung Hee University, 1732 Deogyeong-daero, Giheung-gu, Yongin, Gyeonggi-do, 17104 Republic of Korea; 2 Department of Mechanical Engineering, Gachon University, 1342 Seongnam-daero, Sujeong-gu, Seongnam, Gyeonggi-do, 13120 Republic of Korea; 3 Department of Materials Science and Engineering, Seoul National University, Seoul, 08826 Republic of Korea; 4 Department of Mechanical Design Engineering, Kumoh National Institute of Technology, 61 Daehak-ro, Gumi, Gyeongsangbuk-do, 39177 Republic of Korea

**Keywords:** Sensors, Iontronics, Soft materials, Biomimetics

## Abstract

Many natural organisms have evolved unique sensory systems over millions of years that have allowed them to detect various changes in their surrounding environments. Sensory systems feature numerous receptors—such as photoreceptors, mechanoreceptors, and chemoreceptors—that detect various types of external stimuli, including light, pressure, vibration, sound, and chemical substances. These stimuli are converted into electrochemical signals, which are transmitted to the brain to produce the sensations of sight, touch, hearing, taste, and smell. Inspired by the biological principles of sensory systems, recent advancements in electronics have led to a wide range of applications in artificial sensors. In the current review, we highlight recent developments in artificial sensors inspired by biological sensory systems utilizing soft ionic materials. The versatile characteristics of these ionic materials are introduced while focusing on their mechanical and electrical properties. The features and working principles of natural and artificial sensing systems are investigated in terms of six categories: vision, tactile, hearing, gustatory, olfactory, and proximity sensing. Lastly, we explore several challenges that must be overcome while outlining future research directions in the field of soft ionic sensors.

## Highlights

- This review provides an overview of recent developments in soft ionic sensors inspired by biological sensory systems, focusing on their material properties and working principles.
- The features and working principles of natural and artificial sensing systems are investigated in terms of six categories: vision, tactile, auditory, gustatory, olfactory, and proximity sensing.
- The challenges encountered in developing soft ionic sensors and the future research directions to overcome these issues are discussed.

## Graphical abstract

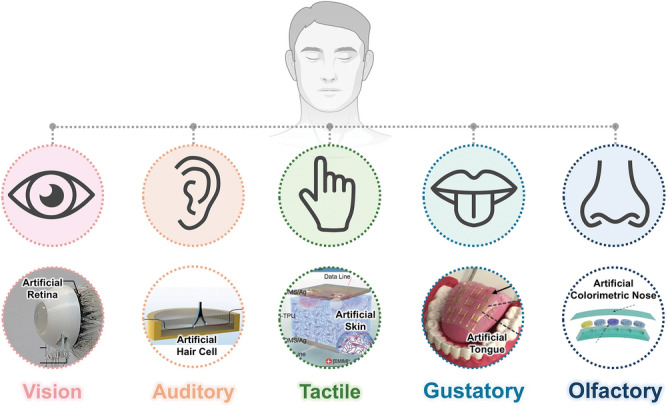

## Introduction

As a result of millions of years of evolution based on natural selection, many natural creatures have optimized and refined various abilities to detect changes in diverse environments. For example, humans have evolved biological sensory systems comprising the five primary senses of sight, touch, hearing, taste, and smell, which all enable us to interact with the world in certain ways. These senses are, respectively, facilitated by specialized sensory organs: the eye, ear, skin, nose, and tongue. These organs use specialized receptors to perceive and convert external stimuli from the environment. The diverse types of external stimuli are converted by biological receptors into visual, mechanical, or electrochemical signals. The biological sensory systems conducting these signal conversions are known for their remarkable adaptability and high sensitivity to external environmental factors [1]. Moreover, the sensory systems are capable of conducting various functions within a single organ [2]. With remarkable and optimized characteristics, the biological sensory systems have inspired humans to replicate their own sensory systems [3–6].

Consequently, recent developments in electronics have led to the creation of artificial sensors mimicking those sophisticated human sensory systems [7–11]. However, replicating the intricate sensory systems remains a significant challenge. The rigid and electron-based materials, which are employed for conventional sensors, are unsuitable for bioinspired sensors. The conventional rigid sensors are incapable of deformation, thus restricting their use on irregularly deformable surfaces. To meet the issue, soft ionic materials have been proposed, endowing both flexibility and stretchability [12, 13]. In addition, their inherent ionic conductivity enables them to replicate the working principle of signal transmission within biological sensory systems. With these strategies, artificial bioinspired sensors have been introduced, transmitting external stimuli into electrical signals with high sensitivity, efficient power consumption, and fast response times [14–18]. Numerous advancements in biomimetics have been achieved that show impressive potential utility in wearable device [19, 20], human–machine interfaces [21–23], artificial organs [24], and so on.

**Fig. 1:**
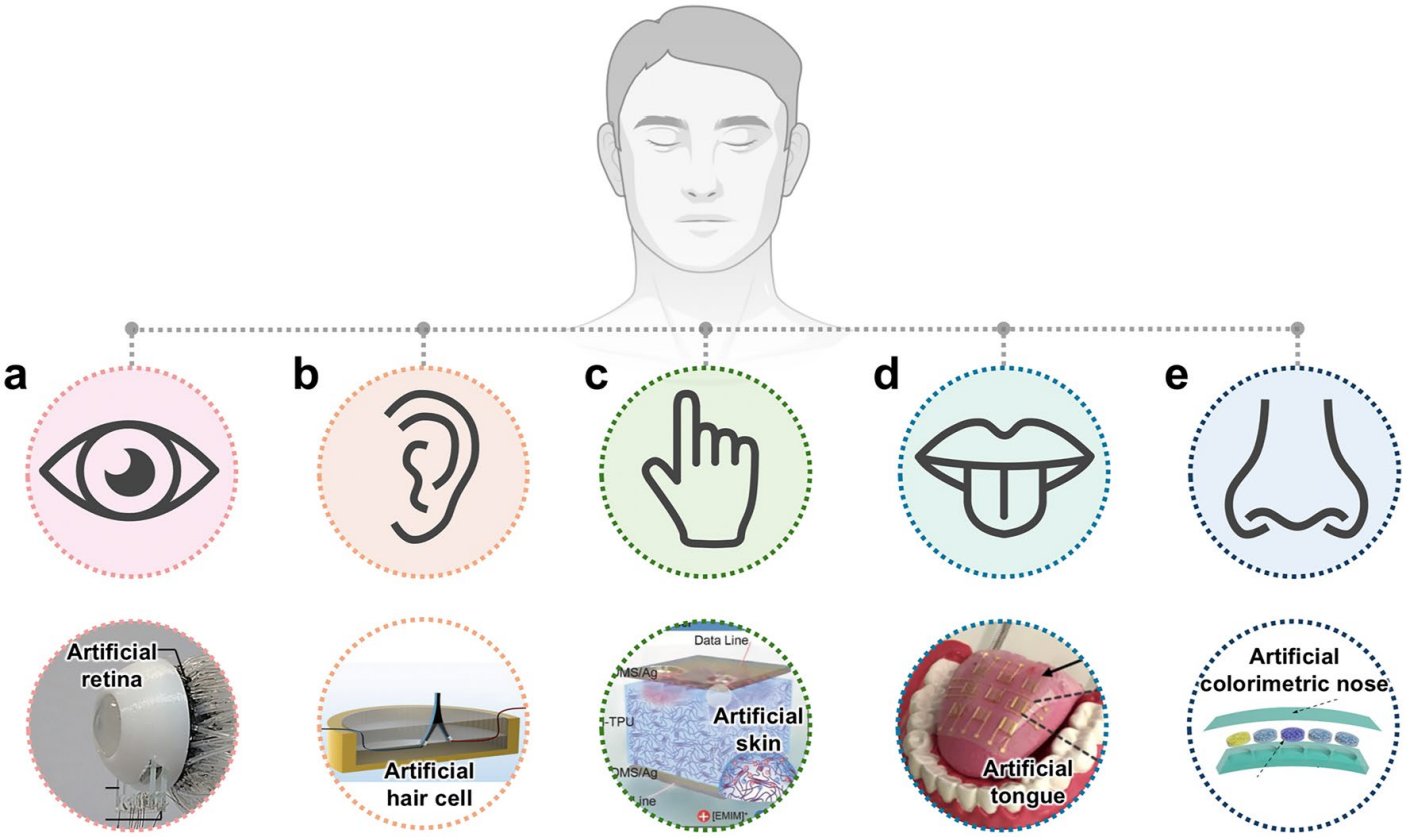
Soft ionic sensors inspired by human. The various applications of such sensors have been demonstrated by emulating the sensing principles and structural characteristics of the human sensory organs, including **a** eyes, **b** ears, **c** skin, **d** tongue, and **e** nose. **a** is reprinted with permission [25]. Copyright 2020, Springer Nature. **b** is reprinted with permission [26]. Copyright 2021, American Chemical Society. **c** is reprinted with permission [27]. Copyright 2017, Wiley–VCH. **d** is reprinted with permission [28]. Copyright 2020, American Association for the Advancement of Science. **e** is reprinted with permission [29]. Copyright 2020, Wiley–VCH

Numerous soft ionic sensors have been designed to imitate the unique human sensory system [26–30], particularly by receiving stimuli and converting them into chemoelectrical signals, thus allowing for interactions with the environment (Fig. 1). Sensors designed to emulate human sensory systems can be categorized into three functional types based on photo [25]/mechano [31–33]/thermo [34, 35]/chemoreceptor [36]. First, an artificial eye, mimicking photoreceptors that detect external light and convert it into visual images, is presented (Fig. 1a) [30]. Next, mechanoreceptors react to external mechanical stimuli, including pressure, vibration, and acoustic waves [37]. Inspired by the mechanoreceptors of the human sensory system, artificial hair cell ear (Fig. 1b) [26, 37] and artificial skin (Fig. 1c) [27] are designed to detect sound and pressure, respectively. Not only the mechanoreceptors, but the thermoreceptors have also provided the inspiration for artificial skin with a wider temperature detection range than that of human skin [38, 39]. Lastly, the chemoreceptors of the human gustatory and olfactory system have inspired the creation of an artificial tongue (Fig. 1d) [28] and a colorimetric nose sensor (Fig. 1e) [29] to detect target chemical substances.

**Fig. 2:**
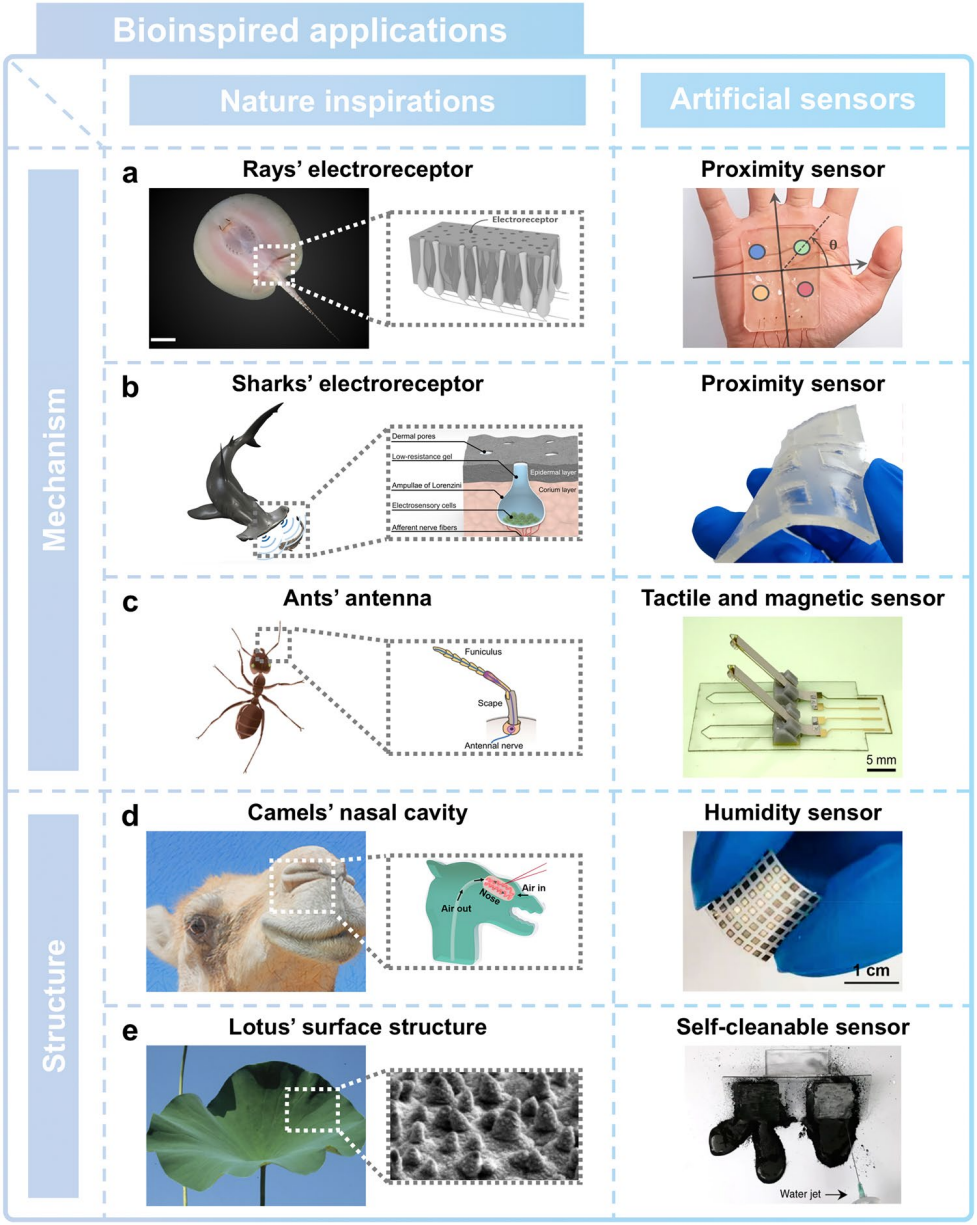
Overview of nature-inspired soft ionic devices. Various sensing applications are inspired by natural organisms, including **a** ray, **b** shark, **c** ant, **d** camel, and **e** lotus. The unique characteristics of soft ionic materials in sensors are capable of realizing the particular working principle of each organism, such as rays’ proximity sensing, sharks’ proximity detection, ants’ tactile and magnetic sensing, camels’ humidity detection sensing, and the structural characteristics of the lotus's superhydrophobic surface. **a** is reprinted with permission [40]. Copyright 2021, American Association for the Advancement of Science. **b** is reprinted with permission [41] Copyright 2022, American Association for the Advancement of Science. **c** is reprinted with permission [42]. Copyright 2024, Springer Nature. **d** is reprinted with permission [43]. Copyright 2022, American Chemical Society. **e** is reprinted with permission [44–46]. Copyright 2009, Elsevier, Copyright 2005, Springer Nature, Copyright 2018, Springer Nature

The evolutionary traces of natural organisms provide researchers with innovative insights into the development of soft ionic sensors and the enhancement of sensing performance (Fig. 2) [40–44]. For example, the proximity sensing capability to detect prey and localize position is a significant capability that facilitates the exploration of natural environments [47]. The proximity sensing capability found in rays (Fig. 2a) [40] and sharks (Fig. 2b) [41] provides creative inspiration for designing proximity sensors. The antennae sensory system of ants (Fig. 2c), which detects pressure, vibration, and magnetic and chemical stimuli, shows potential for multifunctional sensing applications [42]. The remarkable structural features observed in nature have also inspired the novel design of sensors with unique characteristics [48–52]. The structural features of the camel’s cavity (Fig. 2d) have been incorporated into sensors to achieve enhanced humidity sensitivity [43]. The hydrophobic characteristics of lotus leaves have provided the surfaces of such leaves with a self-cleaning capability (Fig. 2e) [44–46]. These structural features provide sights that can help improve sensitivity, broaden the range of sensing targets, and provide multifunctionality. These characteristics of the natural organisms have been integrated with soft ionic materials in various applications, where they have been shown to boost the performance and utility of human–machine interfaces.

**Fig. 3:**
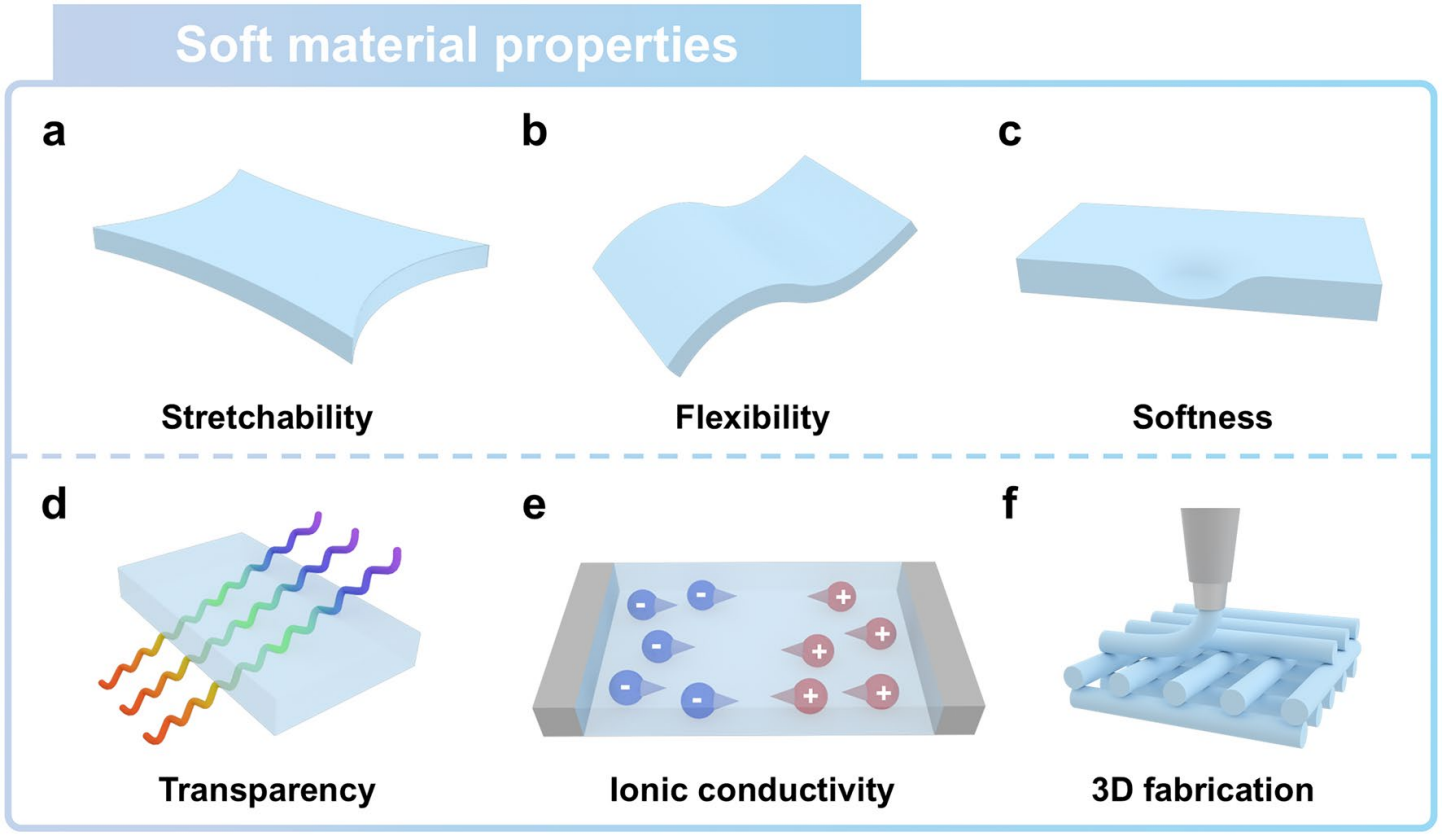
Outstanding properties of soft ionic materials. **a** Stretchability, **b** flexibility, **c** softness, **d** transparency, and **e** ionic conductivity of soft ionic materials facilitate a wide range of applications, including soft ionic sensors. **f** Cost-effectiveness and tunable curing properties of soft ionic materials allow them to be easily fabricated in a three-dimensional structure

Soft ionic materials offer numerous advantages for biomimetic device design [53, 54]. For example, conventional sensors, which generally consist of rigid materials, have relatively poor adaptability to substrate deformation, resulting in deteriorating seamless human–machine interaction. The high Young’s modulus of rigid materials presents challenges in mimicking materials, particularly in replicating the substances found in natural organisms. Meanwhile, soft ionic materials exhibit characteristics originating from their low modulus, such as stretchability (Fig. 3a) [55], flexibility (Fig. 3b) [56, 57], and softness (Fig. 3c) [58]. These properties of soft materials enable them to mimic the capability of natural organisms adapt to ambient environments. Their high transmittance facilitates the transmission of optical information through the materials, paving the way for advancements in transparent electronics. (Fig. 3d) [59, 60]. By attaching to the skin, transparent electronics can conduct medical tasks, providing visual information about skin conditions [61–63]. Polar liquids, such as water and organic liquids, filled into the polymer network of the material readily dissolve ions, thus endowing the ionic materials with ion conductivity (Fig. 3e) [64]. The relatively low cost of water and organic solvents makes it possible to fabricate ionic materials both in large volume and in three dimensions (Fig. 3f) [65]. These fascinating characteristics of soft ionic materials make them particularly suitable for biomimetic applications and human–machine interfaces [66–68].

**Fig. 4:**
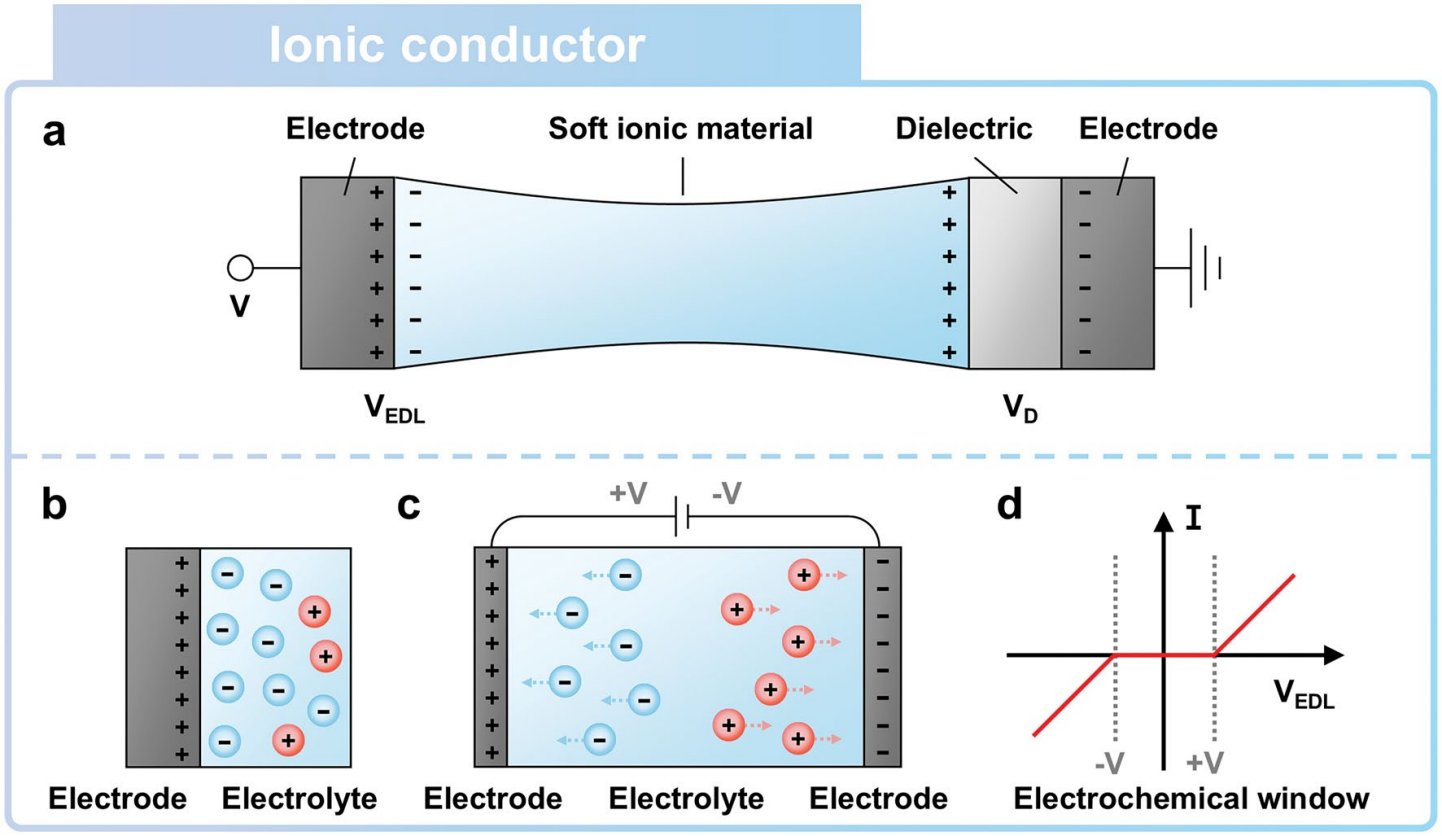
Working principle of soft ionic material as an ionic conductor.** a** Ionic conductor and a dielectric layer are connected in series [59]. **b** Electrical double layer (EDL) is formed at the interface of anions (or cations) in electrolyte and holes (or electrons) in electrode. **c** Both cations and anions migrate through the electrolyte, thus generating ionic currents under input voltage. **d** EDL prevents the migration of ions and electrons, thus inhibiting electrochemical reactions within the electrochemical window

Iontronics, which has advanced as a sophisticated technology, bridges rigid electronic devices and soft biological systems by controlling ions as charge carriers [69, 70]. As a representative material in iontronics, hydrogel has been demonstrated as transparent and soft electronics, including sensors, due to its high stretchability and ionic conductivity [44, 71]. Furthermore, compared to silver nanowires and carbon nanotubes, soft ionic materials such as hydrogel exhibit more stability of resistance under equal stretchability. With these notable advantages, ionic materials-based device can be utilized as ionic conductor (Fig. 4a) [59]. Soft ionic materials can serve as electrolytes, forming the electrical double layer (EDL) at the interface between the electrode and the electrolyte (Fig. 4b) [72]. In ionic materials, both cations and anions are transmitted toward each electrode by voltage difference (Fig. 4c). The formation of EDL prevents electrochemical reactions at the interface, while it also allows electric potential to be transmitted to an external circuit. Devices using ionic materials can operate with electrochemical stability within the applied voltage range of about 1 V across the EDL (Fig. 4d) [59, 73]. The milestone research on soft ionic materials has contributed to the development of soft ionic material-based devices, including bioinspired soft ionic sensors.

**Fig. 5:**
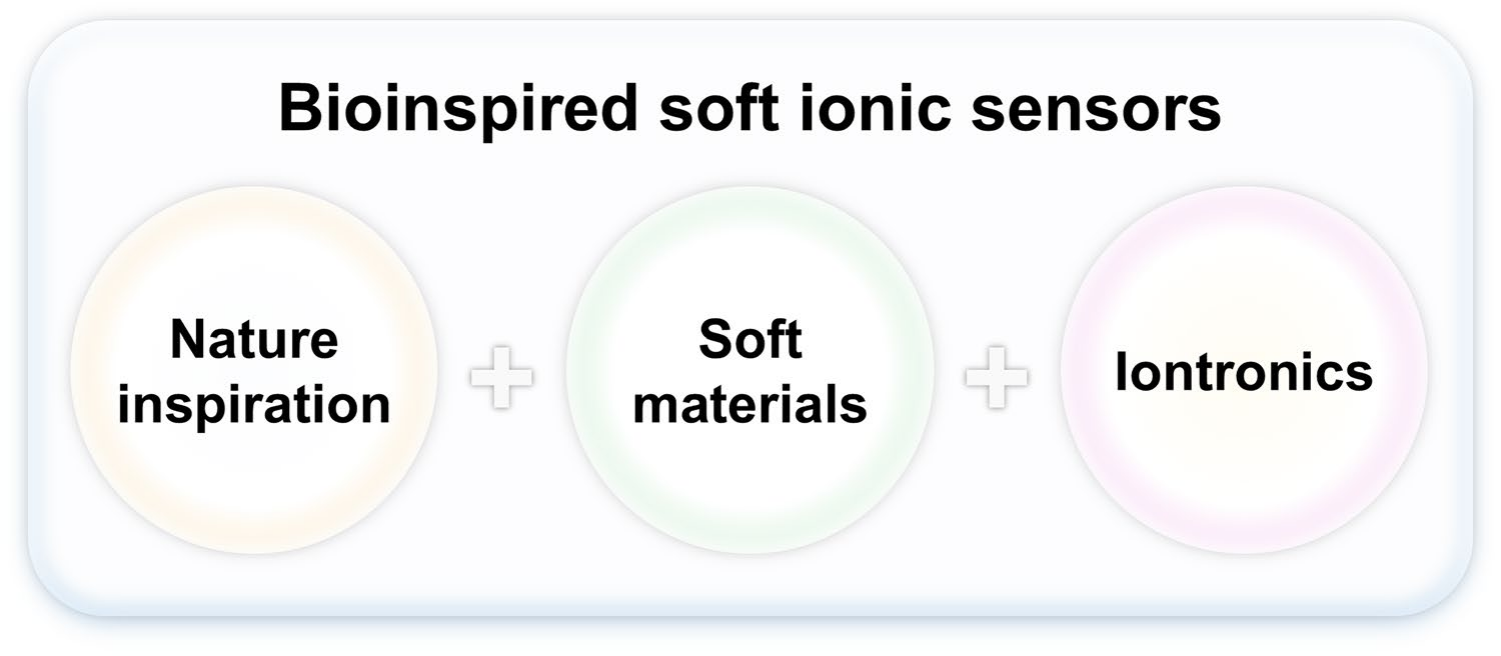
Features of nature-inspired soft ionic sensors. The three promising fields of soft materials, nature inspiration, and iontronics have led to various advancements in the field of sensors. The ionic conductivity and flexibility of soft materials lead to the observed improvements in nature-inspired soft ionic sensors

In this review, we discuss nature-inspired soft ionic sensors, with a focus on their features of evolved natural sensory systems, biological sensing mechanisms, and various applications. The biological sensing principles are analyzed while focusing on four key receptors: mechanoreceptors, thermoreceptors, chemoreceptors, and photoreceptors. Cross-disciplinary advancements in soft materials and iontronics have provided new insights into nature-inspired soft ionic sensors (Fig. 5). From the perspective of human sensory systems, we present an overview of nature-inspired soft ionic sensors, including vision, tactile, auditory, gustatory, olfactory. We extend diverse range of artificial sensors to include proximity sensors inspired by the electroreception capabilities of natural organisms, which are distinct from the five basic human senses [30, 31, 59]. We will explain the biological sensing mechanisms of these sensors, along with an understanding of sensory systems. Finally, we discuss several challenges and propose strategies for real-world applications.

## Nature Inspired Sensor

The functionality of receptors in the human sensory organs has been replicated by a diverse range of artificial sensors [74–76]. Human receptors possess a detection performance range that includes wavelengths from 390 to 750 nm, can detect both low pressure (< 10 kPa) and medium/high pressure (10–100 kPa) regimes, and can discriminate over 1 trillion odors [77, 78]. A diverse range of bioinspired sensors focus on mimicking these specialized functions of human sensory organs by detecting and transducing information from their external environment [79–82]. These artificial sensors have been classified based on the five traditional human sensory organs: skin [83], ear [26], tongue [84], nose [85], and eye [86]. This section will discuss their biological features and scientific applications while focusing on various receptors.

### Artificial Photoreceptors Inspired by Eyes

**Fig. 6:**
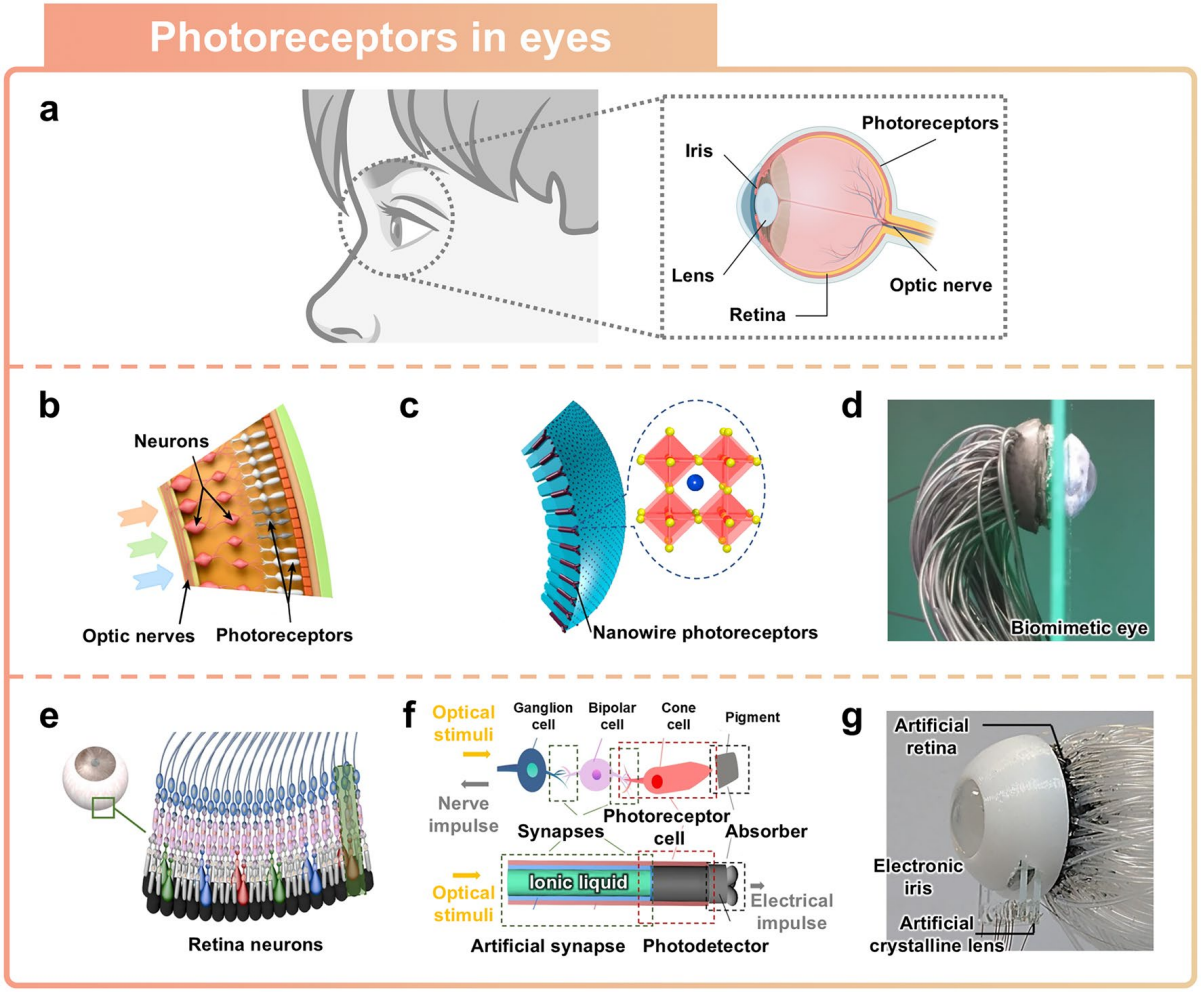
Eye-inspired sensors. **a** Schematic illustration of the human eye structure. **b-d** Artificial retina inspired by rounded shape of human retina. Reprinted with permission [25]. Copyright 2020, Springer Nature. **b** Schematic of the human retina with nerve fibers, neurons, and photoreceptors. **c** Structural representation of an artificial retina fabricated with perovskite nanowires acting as photoreceptors. **d** Photograph of nanowire-interconnected biomimetic vision device. **e**–**g** Artificial photoreceptor distinguishing colors. Reprinted with permission [30]. Copyright 2023, Springer Nature. **e** Schematic illustration of the photoreceptor structure of the human eye. **f** Schematic illustration of the biomimetic neuron structure. **g** Photograph of artificial retina device

The human visual system is the most important organ for interacting with and responding to changes in the surrounding environment, as it perceives approximately 80% or more of the external information perceived by humans [87, 88]. Photoreceptors, which are responsible for perceiving external light and converting it into visual signals and transmitted to the brain via the optic nerve fibers, are arranged along the hemispherical structure of the retina (Fig. 6a) [89]. Various functions of the visual system have been successfully replicated through light-sensitive semiconductor devices, such as photodetectors [90]. While conventional visual sensors have replicated light perception in a planar form, it remains difficult to replicate the spherical structure of the eye. This section will review artificial eyes that replicate structural features of the human eye and utilize ionic materials for visual signal transmission.

The photoreceptors and optic nerves in the human retina are densely arranged, which enables high-resolution image perception of our surroundings [91–93]. Photoreceptors on the human retina can detect light and convert it into neuroelectric signals, which are then transmitted to the brain via the optic nerves (Fig. 6b) [25]. The rounded shape of the retina reduces the aberrations that can occur when light reaches the curved focal plane [94]. An artificial eye employing perovskite nanowire to successfully replicate the functions of photoreceptors and nerve fibers (Fig. 6c). At the interface between the nanowire and the ionic liquid containing electrolytes, an electric double layer is formed. When exposed to light, the nanowire generates electron–hole pairs, with photo-generated electrons migrating toward the interface and holes moving in the opposite direction. These electrochemical reactions, which are facilitated by the segregation of electron–hole pairs, can be detected through individually connected photodetectors and processed in parallel [95]. Consequently, the electrical signals that are transmitted through the nanowires are reconstructed into images via the mimicked artificial eye device. The artificial eye was designed with numerous bundles of nanowires, with the wires aligned at regular intervals using a PDMS socket. (Fig. 6d) The fabricated artificial eye system exhibits a response time of 32.0 ms and a recovery time of 40.8 ms. Compared to the human eyes, which have response and recovery times ranging from 40 to 150 ms, the artificial eye demonstrates equivalent or superior image sensing performance [96].

It is difficult to design a vision sensor with high color selectivity while also achieving high resolution [97–100]. Photoreceptors, specifically rods and cones that detect light and distinguish colors, are densely clustered in the retina (Fig. 6e) [30]. Cones detect and convert absorbed light into electrical signals, which results in color recognition in the brain [101]. The color recognition function of cones has been mimicked by developing a single-cone unit that perceives red, green, and blue light utilizing ionic liquids-based nanowires. Mimicking the structure of retinal neurons, the nanowire features a SnO_2_/NiO double-shell structure that encapsulates an ionic liquid core and a NiO single-shell structure that surrounds a CsPbI_3_ core, thus forming interfaces at the boundaries (Fig. 6f). When light is projected onto the nanowire, longer wavelength light (such as red and green) can penetrate deeper, thus leading to the generation of electron–hole pairs inside the nanowire. The generated carriers are balanced by TFSI^−^ ions in the ionic liquid, which results in gradual variations in photocurrent. Meanwhile, short-wavelength blue light produces a positive photocurrent, with each of the three different types of light exhibiting different photocurrent amplitudes. The artificial eye replicated the structure and function of the human visual system by integrating adaptive optics (Fig. 6g). The adaptive optics configuration consists of a liquid crystal lens for focal length adjustment through an external electric field along with an artificial iris that regulates light intensity by adjusting its transparency. This developed device has optical sensitivities of 400, 106, and 8.59 μW cm^−2^ for RGB colors, and color selectivity can be enhanced by applying − 0.3, 0, and 0.5 V to facilitate efficient charge flow. The artificial lens controls the focal length from 25 cm to infinity within 16 ms, while the electronic iris can adjust the aperture size from 3.14 to 78.5 mm^2^ within 13 ms. These measurements demonstrate fast response times and sensitive color discrimination performance under different light conditions faced by an artificial eye.

### Artificial Mechanoreceptors Inspired by Ears

**Fig. 7:**
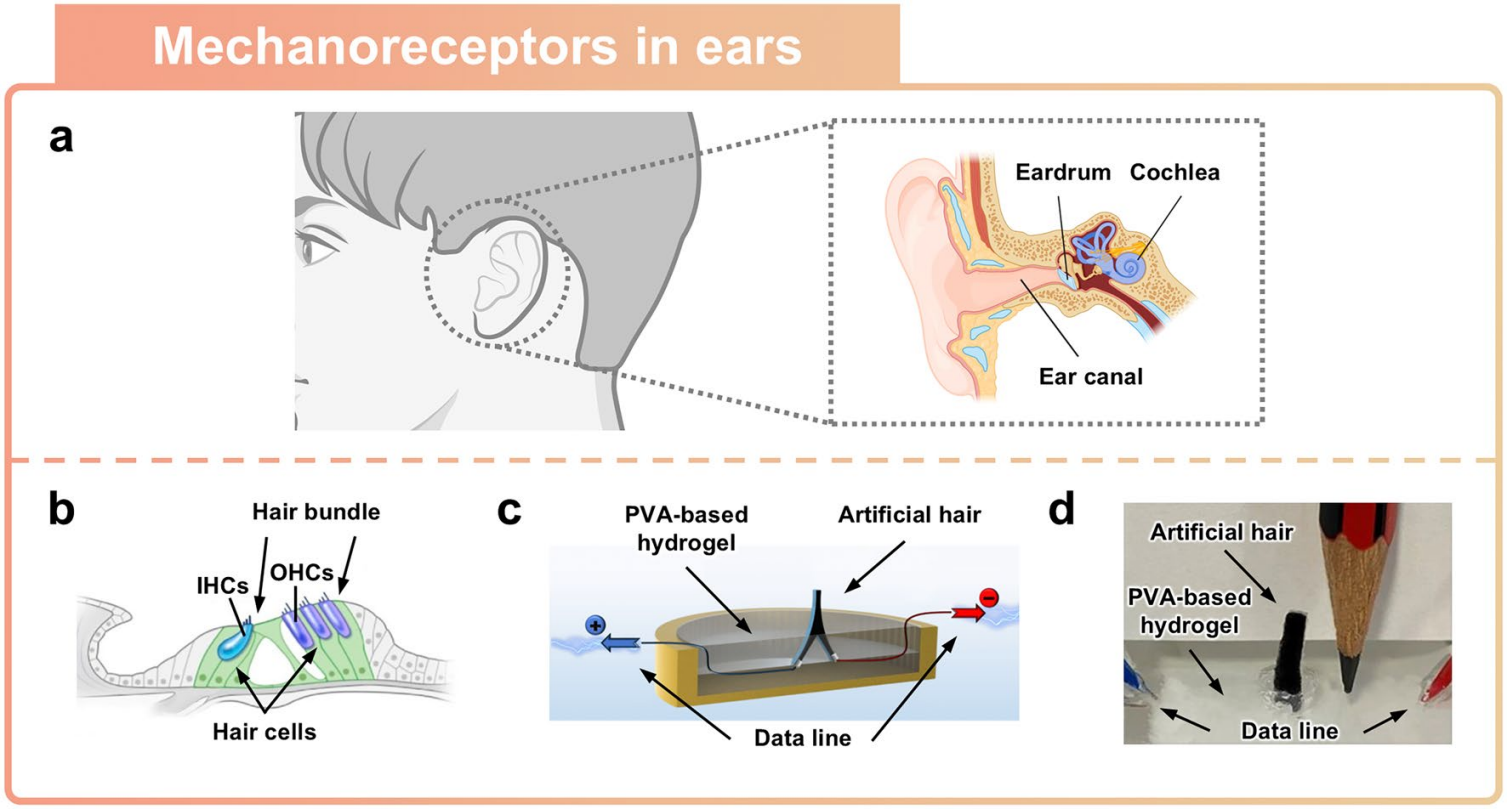
Ear-inspired sensor. **a** Schematic illustration of the human ear structure. **b–d** Piezoresistive-based hair cell auditory sensor. **b** Schematic of the human ear structure showing hair bundles and hair cells. Reprinted with permission [106]. Copyright 2015, The Company of Biologists Ltd. **c** Schematic of piezoresistive-based artificial hair cell auditory sensor. Reprinted with permission [26]. Copyright 2021, American Chemical Society. **d** Demonstration of soft material-based artificial auditory sensor inspired by hair cell structure

Humans can perceive sounds within the frequency range of approximately 20–20,000 Hz, which is known as the human hearing range, through their auditory system [102, 103]. Sound waves that are generated from the external environment induce oscillations in air pressure, which lead to vibrations of the eardrum (Fig. 7a). The air pressure resulting from such sound waves acts as a mechanical stimulus that induce a vibration of the eardrum [104, 105]. The eardrum is affected by the amplitude and frequency of the sound waves, and these vibrations are transmitted to the hair cells of the cochlea, thereby causing mechanical responses. One of the elements of the auditory system, the hair cell, is sensitive to slight vibrations, and it converts transmitted vibrations into electrical signals. The specialized structural features of the evolved auditory system provide humans with the ability to hear a wide range of sound frequencies and to respond to numerous types of potential threats nearby. To implement acoustic sensors that are capable of perceiving a broad spectrum of frequencies, recent studies have utilized functional materials that are sensitive to external and structural designs that amplify faint sounds. This section will cover the principles of the human auditory system in terms of receiving external sounds and explore applications where sensors can be implemented using soft ionic materials.

Hair cells, which are a component of the auditory system, exhibit high sensitivity to vibrations from external sounds and respond to environmental changes via measured sound pressure (Fig. 7b) [26]. Inner hair cells (IHCs) transmit vibrations induced from external sounds into electrical signals, whereas outer hair cells (OHCs) amplify these vibrations [106]. These elements play key roles in the mechanoelectrical transduction of mechanical vibrations, enabling the perception of auditory information. Hydrogels, which are primarily composed of water, are functional materials that can be used to fabricate sensors that are highly sensitive to external sounds which operate in a manner similar to the human auditory system. The structural characteristics of hair cells were replicated using PVA-based hydrogel and vertical graphene nanosheets (VGNs) to implement an artificial auditory sensor (Fig. 7c). Sound waves cause vibrations in the PVA hydrogel, which lead to changes in the electrical conductivity of the VGNs, thus allowing for the detection of sounds ranging from 60 Hz to 20 kHz. The high electrical conductivity of the VGNs is attributed to the carbon base layer and the interconnected graphene walls network, as shown in Fig. 7d. Further, the sensor’s response to acoustic pressure waves in water is quantified by measuring the electrical resistance variation of VGNs.

### Artificial Mechano/Thermoreceptors Inspired by Skin

**Fig. 8:**
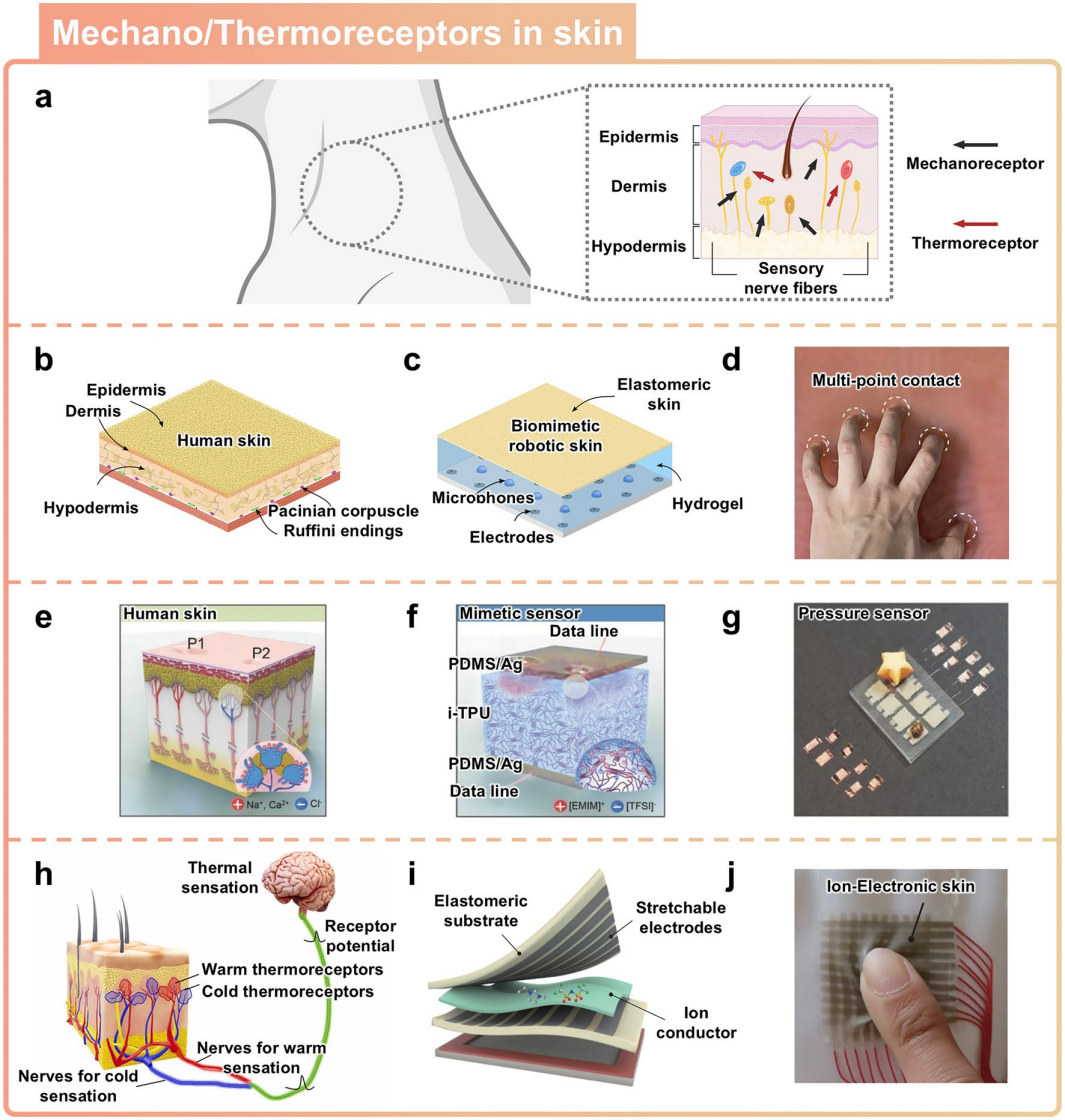
Skin-inspired sensors. **a** Schematic illustration of the human skin with mechano/thermo receptors. **b–d** Skin-inspired elastomeric tactile sensor based on soft ionic material. Reprinted with permission [110]. Copyright 2020, American Association for the Advancement of Science. Schematic representation of multilayer structure of **b** human skin and **c** biomimetic soft ionic skin. **d** Artificial skin capable of detecting multi-touch contact positions. **e**–**g** Tactile sensor inspired by mammalian Merkel cells. Reprinted with permission [27]. Copyright 2017, Wiley–VCH. Schematic representation of receptor structure of **e** human skin and **f** mimetic sensor based on soft ionic material. **g** Demonstration of soft ionic sensor inspired by mammalian Merkel cells that can sense pressure. **h** Schematic illustration of various types of thermoreceptors. Reprinted with permission [39]. Copyright 2022, Elsevier. **i**, **j** Soft ionic multimodal mechano/thermo sensor. Reprinted with permission [111]. Copyright 2024, American Chemical Society. **i** Design of artificial skin with integrated ion conductor. **j** Photograph of artificial skin capable of simultaneously sensing strain and temperature

The skin is the largest organ in the human body, and it functions as a remarkable sensory interface that can acquire various types of vital information about the surrounding environment [107]. Human skin exhibits a complex and highly specialized structure that enables it to detect and respond to a wide range of tactile stimuli [108], including pressure, deformation [37], and temperature changes [109]. The skin’s remarkable sensing capabilities are attributed to the presence of various receptors that are embedded within the intricate architecture of the skin (Fig. 8a). The intricate sensory system of the human skin represents an innovative source that can inspire the development of various types of sensors. This section covers recent developments in soft material-based ionic sensors inspired by the sensory system of the human skin.

#### Artificial Mechanoreceptors Inspired by Skin

The multilayer structure of the human skin, with its embedded mechanoreceptors, protects against a wide range of external forces and threats (Fig. 8b) [110]. Each layer of human skin is specialized and possesses its own distinctive characteristics. Below the epidermis, the dermis provides the skin with resilience and toughness that allows for the effective distribution of forces and pressure [112]. The hypodermis, which is a thin and deformable layer of the skin, can withstand and effectively absorb external pressure [113]. The complex multilayered structure and mechanical properties of human skin have been implemented in multi-touch sensors using hydrogel–elastomer hybrids that exhibit electrical conductivity (Fig. 8c). Silicone elastomers were applied to emulate the mechanical properties of the epidermis, which has a Young's modulus of approximately 2 MPa [114, 115]. The hydrogel is used to replicate the properties of the dermis, which allows it to deform responsively and provide resilience akin to that of skin. The softness of hydrogels with a high-water content transmits vibrations generated by external contact to a large area of the multilayer. When the cross-sectional area of the hydrogel is reduced by external pressure, there is an increase in the impedance values between electrodes. By measuring these changes in impedance value, it is possible to quantify external forces in the range of about 20 N. The elastomer-hydrogel hybrid multi-touch sensor can detect the contact of each finger across wide regions on the surface (Fig. 8d).

In mammalian Merkel cells, the Piezo2 protein has served as a source of inspiration for enhancements to sensor sensitivity (Fig. 8e) [27]. The Piezo2 nanochannel within Merkel cells allows for the movement of cations, thus causing depolarization when external stimuli are applied to the skin [116]. The mechanism underlying the migration of cations has been implemented into a capacitive sensor that uses viscoelastic polymers and ionic fluids. When pressure is applied to the device, ion pairs are pushed toward the densely charged electrode, thus forming a more concentrated EDL. The use of soft ionic materials has allowed for the ion channel functionality to be integrated into capacitive sensors to enhance their sensitivity (Fig. 8f). The sensor, which incorporates ion channel functionality, achieves a high sensitivity of 0.01 pF per kPa at pressures above 10 kPa. Each sensor in the array can detect slight pressures stemming from tiny objects such as a paper star (56.8 mg) or a brown beetle (10.2 mg), as demonstrated in the photographs (Fig. 8g).

#### Artificial ThermoreceptorsIinspired by Skin

Thermoreceptors play a key role in perceiving temperature variations and maintaining the functions necessary for the survival of organisms. The thermoreceptors in the skin are key receptors in the thermosensation of external stimuli. They provide the capability to identify objects and materials and to detect thermal stimuli that are potentially harmful to the body. Thermal stimuli can be divided into four categories, with warm and cool as innocuous conditions and hot and cold as noxious conditions [117]. These thermal stimuli are transmitted as electrical signals through thermoreceptors in human skin (Fig. 8h) [118].

Soft ionic materials can be used as stretchable ionic conductors for temperature sensing based on changes in their ionic conductivity [119, 120]. The change in electrical resistance of stretchable ionic conductors depends on various factors, such as temperature, ion mobility, ion concentration, and polymer network structure. For instance, in thermosensation, an increase in temperature leads to a rise in the diffusion rate of ions, thereby decreasing the electrical resistance of soft ionic materials [121, 122]. The diffusion rate of ions in soft ionic materials can be described with Arrhenius Equation [123]. The Arrhenius Equation ($\sigma ={\sigma }_{0}{e}^{\frac{-{E}_{a}}{RT}}$) describes the temperature dependence of ionic conductivity in polymer electrolyte, where $\sigma$ is ionic conductivity, ${\sigma }_{0}$ is pre-exponential factor, ${E}_{a}$ is activation energy, $R$ is universal gas constant, and $T$ is temperature [124]. Therefore, stretchable ionic conductors are suitable as a key component in temperature sensors due to their ionic conductivity changes caused by temperature variations. To fabricate the multilayer structure of skin, the soft material having ionic conductivity can be integrated as an ionic conductor between two stretchable electrodes (Fig. 8i). The sensing mechanism of artificial skin relies on changes in charge relaxation time based on the ion relaxation dynamics. The charge relaxation time is an insensitive variable to strain, thereby enabling the ion-electronic skin to detect external thermal changes without signal interference induced by various tactile motions (Fig. 8j) [79]. The charge relaxation time consists of two variables, ion conductivity and the dielectric constant. Both variables are intrinsic, which are unaffected by stretching due to the cancellation of dimensional factors. Thus, the charge relaxation time serves as a strain-insensitive variable for measuring temperature changes, even under mechanical stimulations. The artificial receptors are arranged in a 10 × 10 grid array, enabling real-time sensing of contact points, temperature, shear directions, spread, torsion, etc. The performance of the artificial skin was measured with a temperature sensitivity of 10.4% per °C and an average measurement error of 0.29 °C at 50% strain.

### Artificial Chemoreceptors Inspired by Tongue

**Fig. 9:**
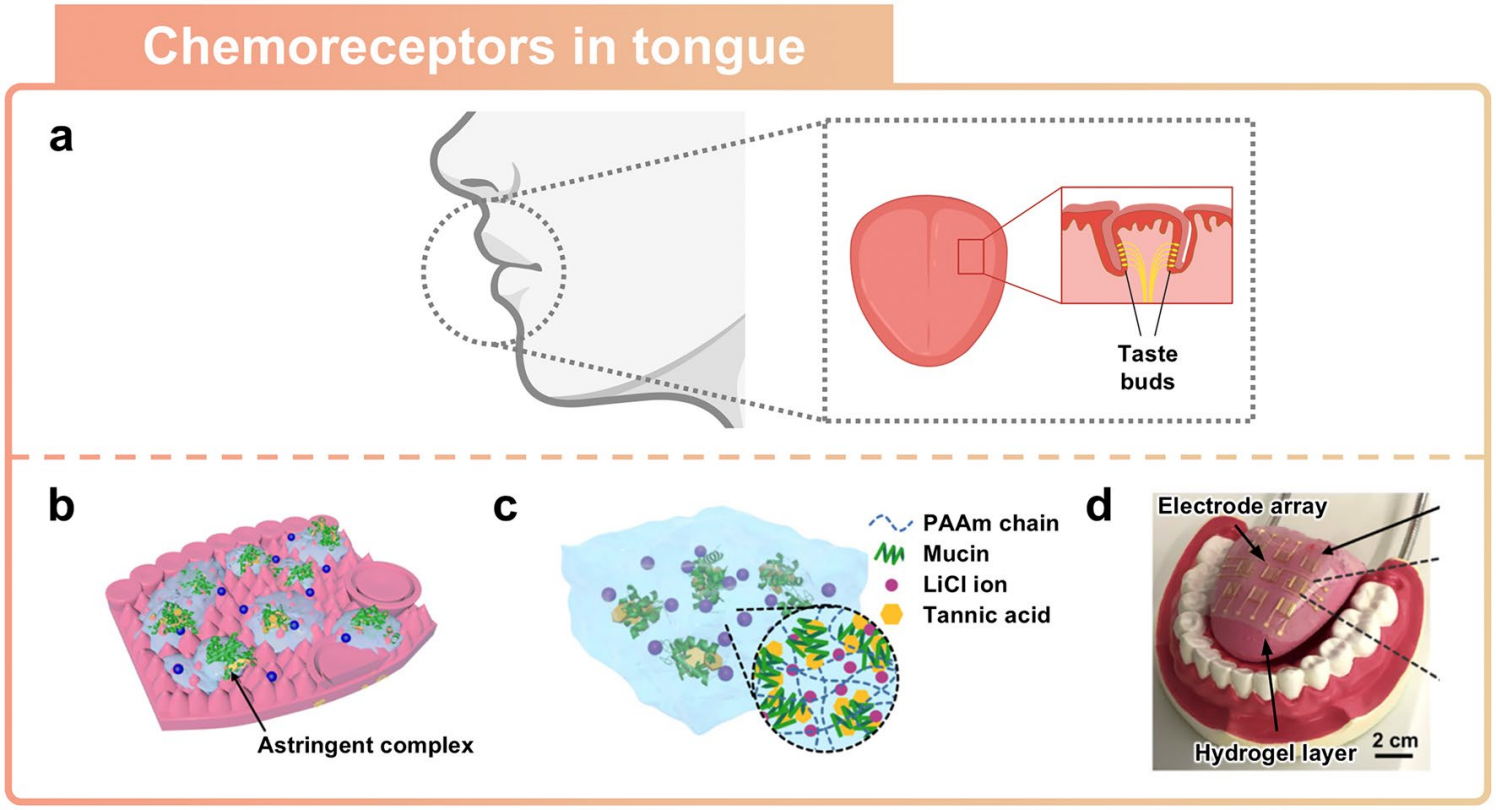
Tongue-inspired sensor. **a** Schematic illustration of the human mouth structure including tongue. **b-d** Artificial tongue imitating the human tongue. Reprinted with permission [28]. Copyright 2020, American Association for the Advancement of Science. **b** Schematic illustration of the structure of the human tongue for taste detection. **c** Working principle of an artificial tongue based on ionic hydrogel. **d** Photograph of an artificial taste sensor emulating the human tongue

The human tongue is an organ that distinguishes taste information using chemical receptors [125–128]. External chemical stimuli are detected by chemoreceptors on the tongue, and these stimuli are then classified into various tastes before being transmitted to the brain (Fig. 9a) [129]. By emulating the human tongue’s gustatory system, as discussed in the previous section with the human nose-inspired sensors, a network of chemical receptors has been implemented using soft ionic materials [28, 84, 130]. This section will present a review of recent developments in artificial tongue sensors using soft ionic materials.

The tongue, which is one of the specialized organs known for its softness, sensitivity, and flexibility, can detect a wide variety of tastes [131]. The human taste sensory system can distinguish between the five primary tastes through the taste receptors located on the tongue [132]. Saliva, which consists mostly of water and electrolytes, is vital for taste perception. The salivary film dissolves taste substances, thus facilitating binding to taste receptors or the transport of ions through ion channels (Fig. 9b) [28]. Taste signals from water-soluble tastants are generated either by depolarization through binding of tastants to receptor cells or by ion transport through the ion channels [133]. Hydrogels, which are largely composed of water and containing electrolytes, can be used as conductive soft ionic materials [60]. To implement an artificial tongue, artificial saliva layer was created using porous hydrogel, and LiCl ions were incorporated as an electrolyte to achieve electrical conductivity (Fig. 9c). When the hydrogel layer is exposed to tannic acid (TA), a type of tannin, the incoming TA molecules bind with mucin, a glycoprotein present in mucus, thus forming hydrophobic aggregates. During this process of complexation, the pore walls tear and the microporous hydrogel structure is transformed into a nanoporous structure. Tearing of the pore walls results in the reconstruction of nanoscale porous structures, which changes the electrostatic interactions between the pore walls and electrolytes. With reduced electrostatic interactions in the hydrophobic nano-pores and electrolytes, Li^+^ and Cl^−^ ions flow more efficiently through the micro/nanoporous structures. As a result, the enhanced ion transport enables a faster response time and a wider sensing range. As shown in (Fig. 9d), the artificial tongue can simultaneously distinguish between different concentrations of TA solution through a 3 × 3 array. Without requiring pre-calibration, the implemented artificial tongue can distinguish molar concentrations from 0.0005 to 1 wt% range and detect tannic acid with high sensitivity in about 10 s.

### Artificial Chemoreceptors Inspired by Nose

**Fig. 10:**
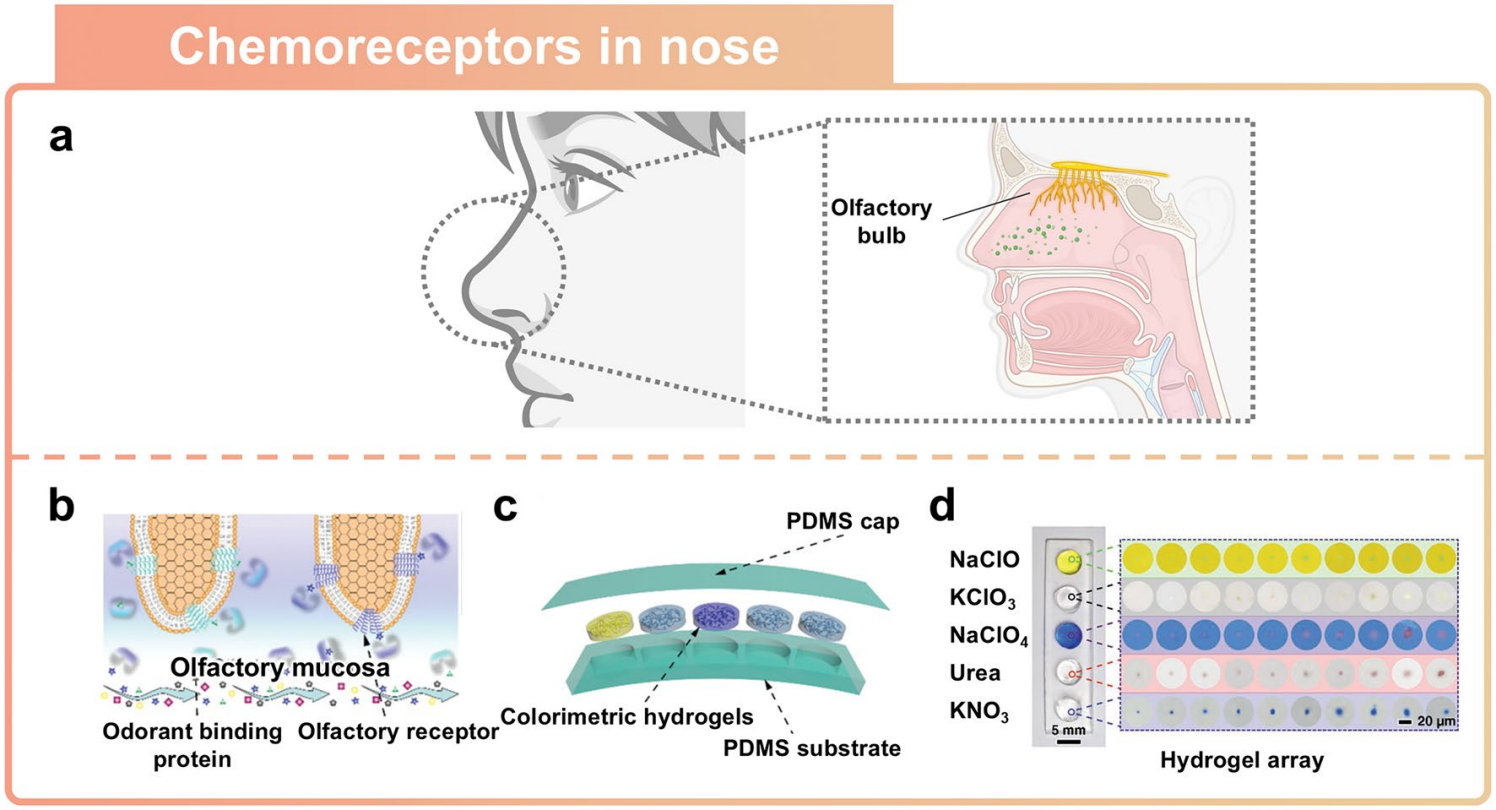
Nose-inspired sensor. **a** Schematic illustration of the human olfactory system. **b-d** Artificial olfactory colorimetric device. Reprinted with permission [29]. Copyright 2020, Wiley–VCH. **b** Schematic representation of a natural olfactory system including olfactory receptors. **c** Structure of an artificial olfactory system based on a colorimetric hydrogel array. **d** Photograph of a nose-inspired sensor exhibiting a color change in response to different types of microparticles

The components of the human olfactory system consist of the human nose and olfactory receptors [134–136]. Chemoreceptors in the human nose are a crucial element of the olfactory system, as they allow for chemical stimuli to be detected from the nearby environment (Fig. 10a) [137]. Transferred chemical information provides a crucial criterion for determining what is either safe or dangerous to the human body. External chemical stimuli are converted into physical and chemical signals, including electrical signals [138], optical signals [139], or other mechanical signals [140], through sensors. This section will explore a novel olfactory sensor that is inspired by the human nose and has been fabricated using soft ionic materials to deliver information to various forms.

Potential airborne hazards are often identified using artificial olfactory sensors [85, 141–143]. The human olfactory system has inspired various advancements in artificial olfactory sensors, which are capable of remotely distinguishing and analyzing odorants. The olfactory mucosa and specific odorant binding proteins in the human olfactory system absorb and diffuse odorants as they interact selectively with target microparticles (Fig. 10b) [144]. Consequently, these chemical reactions are converted into electrical signals by the olfactory receptors in the olfactory bulb, after which they are subsequently transmitted to the brain via olfactory sensory neurons [143]. As a type of artificial nose, a colorimetric nose is capable of distinguishing different odorants and providing optical signals in a wide range of colors. Hydrogels, which are primarily composed of water, can be used as biomimetic materials in olfactory sensors mimicking the function of the olfactory mucosa in the human olfactory system [145]. The water in the hydrogel is replaced by different ionic liquids or solvents, each of which can react with different substances. It makes hydrogel perform as a manifold containing the colorimetric reagents, similar to the odorant biding proteins in human nose [29]. Colorimetric hydrogel-based artificial olfactory sensing devices are capable of visualizing optical signals through various color changes in response to different types and concentrations of target chemical substances (Fig. 10c). Polydimethylsiloxane (PDMS) was used as a barrier to prevent the dehydration of the hydrogel and protect it from damage under ambient conditions [146]. The PDMS substrate separates and anchors each hydrogel, thereby replicating the wide range of chemical discrimination abilities of the human olfactory system. The colorimetric hydrogel array can selectively react with specific hazardous target chemical substances such as NaClO, KClO_3_, NaClO_4_, urea, and KNO_3_ (Fig. 10d). Under ambient conditions, the hydrogel physically captures airborne microparticulates onto its surface. The captured microparticulates are absorbed and diffused into the hydrogel's liquid. It leads to chemical reactions with the colorimetric reagents embedded in the liquid. For instance, the detection of hypochlorite, a chemical compound that provides oxygen in an explosive reaction, is based on Berthelot’s reaction [147]. During this process, the generated indophenol blue dye visualizes the concentration through a color change. A hydrogel-based colorimetric device exhibits sensitive sensing capabilities that mimic the human olfactory system. This device can discriminate microparticulates with diameters ranging from 1.2 to 9.8 μm as well as a minimum mass of 39.4 pg.

### Artificial Electroreceptors Inspired by Ray

Beyond the previously discussed conventional five human senses, some species pose unique proximity sensing capabilities [41, 47, 148, 149]. These sensing capabilities have evolved to allow such species to locate prey and avoid predators. In aquatic environments, elasmobranchs can detect electric fields emitted by prey through their electroreceptors, referred to as Ampullary receptors [150]. Ampullary electroreceptors consist of a cutaneous pore located on the skin and a canal filled with an electrically conductive gel [151]. External electric stimuli trigger the opening or closure of voltage-gated ion channels in electrosensory cells located at the terminal end of the canal. These electrosensory systems enable elasmobranchs to detect the intensity of electric fields and provide evolved predatory functions optimized for aquatic environments. In recent years, researchers have developed various types of artificial proximity sensors inspired by these natural electroreception sensing abilities.

**Fig. 11:**
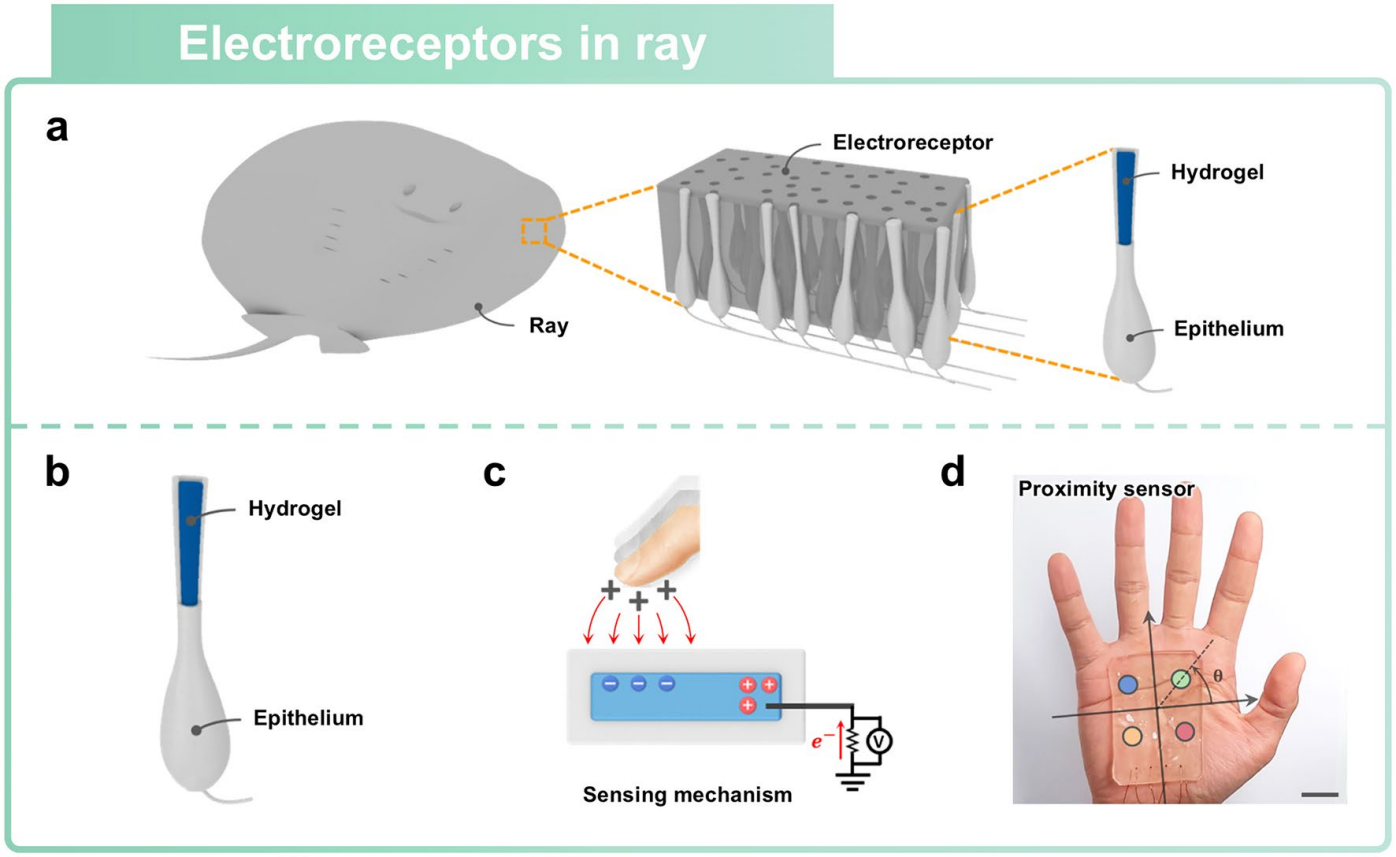
Ray-inspired proximity sensor. **a-d** Artificial proximity sensor inspired by ray. Reprinted with permission [40]. Copyright 2021, American Association for the Advancement of Science. **a** Network of electroreceptors of Ray. Rays have electroreceptors beneath their skin, which allow them to perform proximity sensing and perceive adjacent information. **b** Schematic representation of artificial electroreceptor structure inspired by ray. **c** Working principles of proximity sensor by measuring induction of voltage from external electric fields. **d** Demonstration of proximity sensor based on soft ionic materials

Rays, which are a type of elasmobranch fishes, exhibit proximity sensing capabilities that allow them to detect prey underwater [152]. Their sensing capabilities are realized by a network of electroreceptors embedded beneath their skin (Fig. 11a) [40]. Rays’ electroreceptors detect changes in electric fields that are caused by the biomechanical activity of prey creatures. The electrosensory systems of rays allow them to locate target prey without having to make physical contact. Inspired by this proximity capability of rays, an artificial electroreceptor was introduced. This electroreceptor consists of a hydrogel that receives electric fields and an epithelial layer that encapsulates the core, thus allowing the intensity and polarity of electric fields to be sensed (Fig. 11b). The relative positions of prey can be estimated by comparing the electric field intensities that are detected by each electroreceptor in the network. The proximity sensing principle of rays has been successfully replicated through the induction of voltage from external electric fields (Fig. 11c). When a charged object approaches the artificial electroreceptor, surface static charges on the object induce a voltage in the hydrogel. The movement of ions accumulated in the hydrogel generates an ionic current that is proportional to the intensity of the electric field [153, 154]. The ion mobility in the hydrogel contributes to the transmission of the electric field to connected data line, resulting in the generation of a voltage signal [70]. Therefore, this signal measurement of induced voltage allows the sensor to detect proximity without having to make physical contact. An artificial transparent network consisting of four individual transparent ionic sensors was demonstrated (Fig. 11d). Each of the four ionic sensors acts as an electric field receiver with which to perceive spatial information. The array of the sensors allows for the estimation of the orientation and relative position of objects by comparing the voltages induced at each sensor.

## Conclusions and Future Directions

In this review, we explore nature-inspired soft ionic sensors, focusing on the unique features of natural sensory systems, working mechanisms, and potential applications. Natural sensory systems exhibit remarkable sensitivity to environmental changes. These sensory systems provide valuable insights for the development of artificial sensors. These nature-inspired sensors have been advanced through integration with research fields of soft materials and iontronics. For example, soft ionic materials with adaptive properties (e.g., volume, resistance, color, and transparency) in response to external stimuli broaden their range of applications in nature-inspired sensors.

We summarized soft ionic sensors into six types based on their sensing targets, performance, materials, and characteristics (Table 1). To compare the performance of soft ionic sensors, key metrics such as sensing range, sensitivity, accuracy, response time, and recovery time were investigated. However, due to the lack of standardized measurement protocols, sensors performance may vary under diverse environmental conditions. For instance, the ionic conductivity of these ionic sensors is affected by environmental temperature, which can be described with the Arrhenius equation [155]. The performance of soft ionic sensors is also affected by relative humidity. Under dry conditions, evaporation can potentially reduce sensor performance due to the loss of solvent molecules within ionic materials. For these reasons, the standardized measurement protocols are required for the quantitative evaluation of soft ionic sensor performance. For instance, sensor performance measurements could be conducted under standard room conditions (26 °C, 50% relative humidity), similar to the one-sun conditions in photovoltaics [156], or the ZT factors utilized in thermoelectric materials [157]. Such conditions provide a guideline for standardized measurements protocols, facilitating reproducibility and ensuring consistent fabrication of soft ionic sensors.

**Table 1:** A summary of the soft material-based sensors

Mimicked organ	Sensing type	Target	Performances				Material	Refs
			Sensing range	Sensitivity/ Accuracy	Response/ Recovery time	Characteristics		
Eyes	Vision	Light (photons)	Focal length: 56 − 61 mm	N/A	N/A	Field of view (FOV): 40˚ Tunable microlens	Liquid crystal elastomer, PDMS	[169]
			N/A	N/A	30 s upon 230 mW cm^−2^ / N/A	Lignt transmission: 10% − 70%	Liquid crystal elastomer	[170]
			Focal length: 1.37 mm	N/A	N/A	Field of view (FOV): 160˚ Radius of curvature (R): 6.96 mm	PDMS	[92]
			Focal length: 16 mm	N/A	19.2 ms / 23.9 ms	Field of view (FOV): 100.1˚ Resolution: 4.6 $\times$ 10^8^ cm^−2^	PDMS	[25]
Skin	Tactile	Deformation (pressure)	0.7 − 20 N	N/A / 98.7% with a machine learning model	N/A	Receptor density: 0.0625 U cm^−2^	PAAm hydrogel, silicone elastomer	[110]
			1.8 Pa − 50 kPa	25.8 nF kPa^−1^ over 10 kPa / N/A	47 / 63 ms	Emulate the Piezo2 nanochannel	TPU ionogel, PDMS	[27]
	Strain	Deformation (strain)	> 600%	N/A	140 / 230 ms	Gauge factor: 3.94	PNIPAM hydrogel	[1]
			> 1860%	N/A	165 / 155 ms	Gauge factor: 1.74 − 3.17	PAAm hydrogel	[171]
Ears	Auditory	Vibration (frequency)	1 − 150 Hz	4121 kPa^−1^ in stress / > 99%	N/A	Gauge factor: 12,787 at strain	Anisotropic conductive gel	[172]
			20 Hz − 2 kHz	153—217 nF kPa^−1^ or 24 uC N^−1^ at a bias of 1.0 V / N/A	N/A	Detecting underwater sound	PAAm hydrogel	[173]
			20 Hz − 3 kHz	900 nF kPa^−1^ / N/A	N/A	Gate-free hydrogel-graphene transistor	PAAm hydrogel-graphene	[174]
			60 Hz − 20 kHz	0.173 mV kHz^−1^ / N/A	N/A	Linearity: maintain sensor linearity up to 1800 mV	PVA hydrogel-graphene	[26]
			10 Hz − 100 kHz	24 mV Pa^−1^ / N/A	N/A	Detect sound underwater from different directions (0 − 90°)	PAA-co-PAAm ionogel	[175]
Tongue	Gustatory	Ammonia	0.2454 − 1.25 ppm	N/A	N/A	Colorimetric hydrogel with adhesive and self-healing properties	PVA hydrogel	[176]
		D(-)fructose (sweetness), NaCl (saltiness), acetic acid (sourness)	0.086 − 0.51 M	N/A / 83.4% with a machine learning model	N/A / < 40 min	Durability: maintain sensitivity after 10 days	Poly(DMAPS-co-HEMA) hydrogel	[177]
		NaCl (saltiness)	0.02 − 6 wt%	N/A	< 1 s / N/A	Durability: retains 10% of taste memory after 1000 s	Chitosan ionogel	[178]
		Monosodium glutamate (MSG), disodium inosinate (IMP)	10^–15^ − 10^–2^ M	N/A	N/A	Detection of umami substances in fermented fish	PAAm hydrogel-carbon nanotube	[179]
		Potential of hydrogen (pH)	4.0 − 7.5 pH	N/A	N/A	Durability: no obvious color changes in 5 weeks	HEAA ionogel	[180]
		Tannin acid (astringency), polyphenol (bitterness)	0.0005 − 1 wt%	0.292 wt%^−1^ / N/A	< 10 s / N/A	Durability: 10 days under 25 °C, 60% relative humidity	PAAm hydrogel	[28]
Nose	Olfactory	Short-chain fatty acids (SCFAs)	0.07 − 1.30 ppm	N/A / < 91.6% with a machine learning model	N/A	Durability: retained 50% functionality after 16 weeks	P(VDF-HFP) ionogel	[181]
		Mixed gas (H_2_, NH_3_, and C_2_H_5_OH)	0 − 1 ppm of NH_3_ & 0 − 50 ppm of H_2_ & C_2_H_5_OH	N/A	N/A	Simultaneous detection of mixed-gas components	P(VDF-HFP) ionogel	[182]
		Viral proteins (H5N1, H1N1, and COVID-19)	0.1 fg mL^−1^ − 10 ng mL^−1^	N/A	< 10 min / N/A	Multi − channel ion − gated transistor	PVA ionogel, PDMS	[183]
		Nitrogen dioxide (NO_2_)	2.66 − 600 ppm	N/A / 81.2% with a machine learning model	N/A	Long-term retention time (19,000 s)	PEGDA Ionogel	[36]
Electro- receptor	Proximity	Electrical field	Distance: > 240 cm	N/A	N/A	Sensing induced voltage: 720 mVpp	PAAm hydrogel	[40]
			Distance: 5 − 90 cm	N/A / > 97% with a machine learning model	N/A	Ionic conductivity: 0.845 S m^−1^	PAAm-co-EG hydrogel, PDMS	[41]

Nature-inspired soft ionic sensors are key components in a wide variety of applications, particularly in human–machine interfaces and the field of soft robotics [158]. The high stretchability and softness of these sensors enable humans to more closely interact with soft robotics, resulting in seamless integration across various wearable device applications [159, 160]. The stretchable sensors also have the potential in biosignal monitoring devices, providing user comfort during daily life [161]. Furthermore, recent advancements have enhanced their versatility, with specific features incorporated into each environment [162]. Biomimetic actuators, as well as sensors, demonstrate immense potential with their material (e.g., thermo-responsiveness, phase change, adhesion, self-healing) [163–167]. As an industrial application, they can be integrated with soft sensors and electronics [168]. For instance, such integration replicates the function of a human hand, allowing the adaptive grasping of various shapes and sizes.

**Fig. 12:**
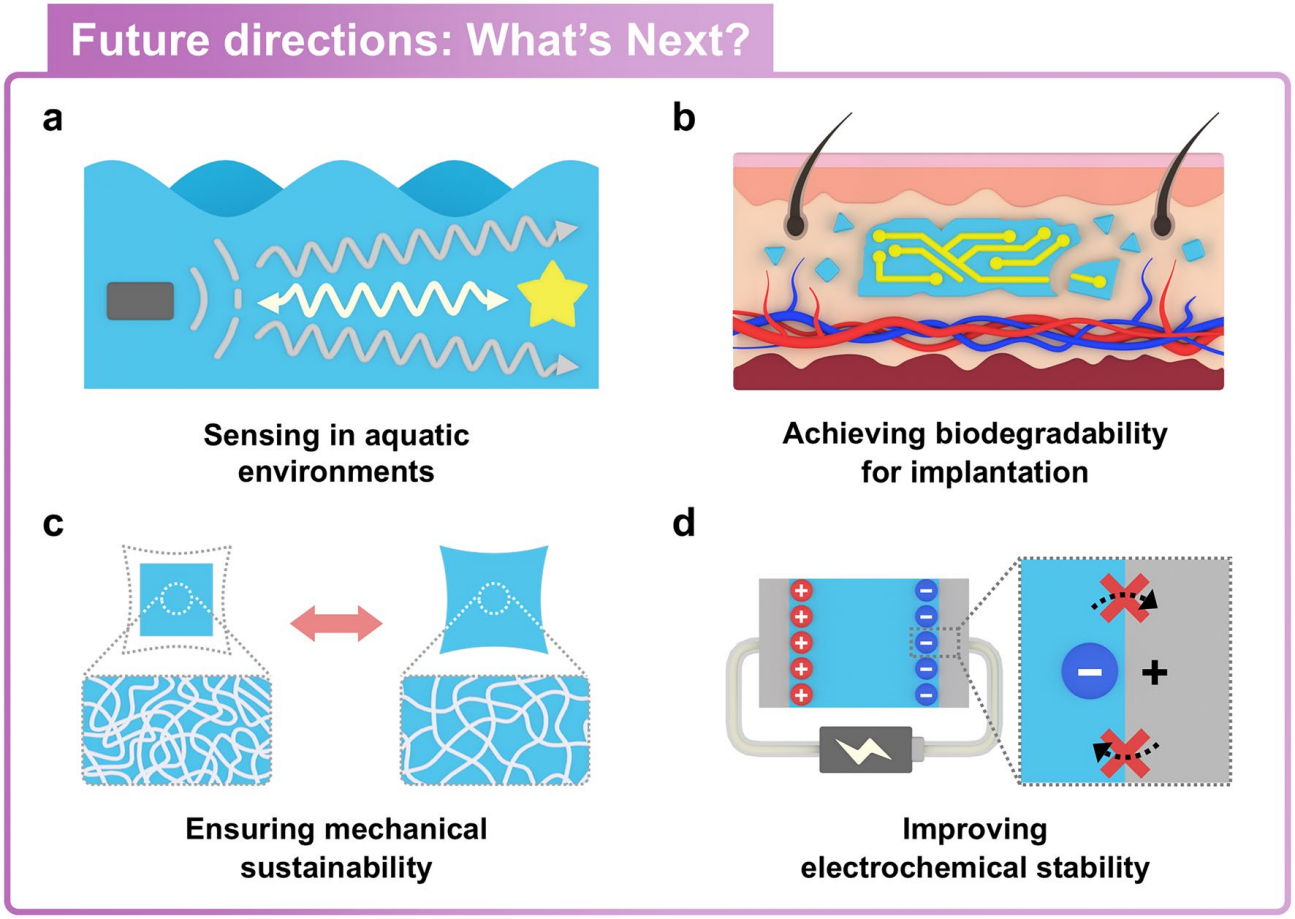
Schematic of future research directions for soft ionic sensors. **a** Sensing in aquatic environments, **b** achieving biodegradability for implantation, **c** ensuring mechanical sustainability, and **d** improving electrochemical stability are several remaining challenges to improve soft ionic sensing capability

However, even though previous studies have proven the potential of promising soft ionic sensors, they are still in the early stages and face several challenges before they can be used in daily life. Here, we suggest future research directions in terms of four key perspectives: sensing in aquatic environments, achieving biodegradability for implantation, ensuring mechanical sustainability, and improving electrochemical stability (Fig. 12).

### Sensing in Aquatic Environments

Sensing capability of passive electroreception in aquatic environments has inspired the development of artificial proximity sensors. For instance, some species including rays and sharks locate their prey using electroreceptors densely distributed on their skin [184, 185]. Inspired by these sensing capabilities, artificial proximity sensors can detect the relative distance of an object by measuring changes in electric fields. These proximity sensors are applicable as wearable devices, allowing humans to interact with their surroundings without physical contact. Some of proximity sensors that operate based on detecting changes in an electric field might suffer from reduced sensing capabilities in underwater, as moisture in the body can shield interactions with electric field (Fig. 12a). To address this issue, replicating the dense electroreceptor networks observed in certain species offers an effective approach to improving the sensitivity of proximity sensors. This approach allows proximity sensors to achieve higher spatial resolution, enabling sensitive perception of target positions through the comparison of electric field intensities at each electroreceptor. As another example, the signal processing methods with machine learning present an effective approach in underwater sensing [186]. The feature extraction based on machine learning offers accurate pattern recognition and noise filtering from complex signal data. Therefore, utilizing machine learning techniques could improve the detection of low-level sensory signals and extraction of high-level features in aquatic environments.

### Achieving Biodegradability for Implantation

Conventional wearable devices are commonly employed for monitoring vital signs in humans, yet their low deformability causes discomfort when attached to the skin surface. To address this inconvenience, implantable devices have been proposed. Skin-like soft materials with biocompatibility and a low Young's modulus have shown numerous applications in implantable devices [187–190]. However, several issues are still raised to utilize soft materials in implantable devices. For example, the implantation of devices necessitates surgical insertion and removal, which can cause inevitable pain and potentially leave scar on the skin (Fig. 12b).

To meet this issue, biodegradability is a key property that enables the design of implantable devices within the human body. Biodegradable devices are designed to be naturally eliminated from the body without additional surgical removal after task completion. One of the major challenges in implantable devices is their limited capability to control degradation time in the body. The ability to control the degradation time of implanted devices is necessary to extend their operational period or accelerate their removal in the body. To manage degradation period, endowing stimuli-responsive capability to the biodegradable materials could be one of attractive approaches including external stimuli (e.g., electromagnetic fields, thermal stimuli, or vibrations) [191–193]. Further advancements in functional soft materials with controllable biodegradation will lead to enable significant developments in implantable devices.

### Ensuring Mechanical Sustainability

The low modulus of elasticity of ionic materials allows ionic material-based sensors to easily adapt and conform to external deformations, thus making them suitable for application to human skin. Further, the low level of modulus has contributed to enhancing their pressure and strain sensing capabilities [194]. For instance, soft ionic materials with a low Young’s modulus exhibit high compliance. The compliance of these materials ensures the stability of the interface for attachable devices, even under dynamic deformation of human skin [195]. However, the soft ionic materials must be resilient to maintain consistent sensing performance (Fig. 12b). Repeated and excessive compressive and tensile deformation can cause unendurable volumetric changes in the polymer chain, which can in tern lead to unwanted permanent damage. These deformations can reconfigure polymer networks, potentially deteriorating their intrinsic resilience.

Intense efforts to improve the mechanical properties of soft materials have been reported. The dehydration of soft ionic materials is a significant issue that can occur in ambient conditions. To address this issue, incorporating hygroscopic materials (e.g., LiCl, CaCl_2_, ethylene glycol) could be an effective approach in preventing evaporation [196, 197]. However, these materials exhibit high sensitivity to humidity, which could result in unexpected swelling. The elastomeric encapsulation with chemical adhesion can protect sensor components and prevent evaporation, similar to the natural barrier of fruit peels. For instance, an ionic wire, fabricated by encapsulating conductive gel with a silicone tube, prevents evaporation of soft ionic materials [47].

The synthesis of polymers with double-network structures is an effective strategy to enhance mechanical properties of materials. For example, double-network hydrogel is a soft material that is stretchable and improves its toughness through covalent and ionic cross-linking of the polymer network [60, 198]. The enhanced toughness is achieved by the unzipping of ionic cross-links in the double-network structure, thereby effectively dissipating energy. These double-network hydrogels demonstrate robust recovery capability after deformation caused by mechanical impact. However, excessive external force applied to the hydrogel causes the plastic deformation of polymer networks and inevitable failure. To recover their initial state and thus enhance durability, the self-healing capability has been introduced. The self-healing capability of materials enhances the durability of soft sensors, enabling them to recover their initial functionality for repeated use. The self-healing properties of soft materials, similar to skin, allow them to recover from mechanical damage and maintain consistent sensing performance [53, 199]. Despite their self-healing properties, soft materials may exhibit limitations, such as misalignment or decreased transparency at the healed regions.

In addition, a highly entangled polymer network was also introduced with greatly outnumbered cross-links by entanglements [200]. For example, a hydrogel with this highly entangled network demonstrates enhanced mechanical properties, such as high toughness, resistance to fatigue, stretchability, and compliance, due to the transmission of tension within the polymer network. The entangled networks provide the hydrogel with high elasticity and fatigue threshold (~ 240 J m^−2^), along with a lower friction coefficient due to their longer polymer chains. The hydrogels with entangled networks demonstrate high wear resistance and mechanical stability, maintaining their polymer network in aquatic environments. However, the high Young’s modulus resulting from the dense entanglements inevitably causes lower compliance compared to conventional hydrogels. To address this issue, the synthesis methods for materials to balance the density of cross-links and entanglements could be further explored.

### Improving Electrochemical Stability

Soft ionic materials have replaced conductive components in electronic devices based on their ionic conductivity, where ions are used as charge carriers. Because electron-based conductors are still dominant worldwide, the application of ionic conductors to electron-based devices requires that they be compatible with electronic conductors. A capacitive component called the EDL is formed at the interface between the ionic and electronic conductors, and this component is not present at the interface between electron-based conductors. At the interface, inevitable electrochemical reactions can occur upon the application of voltages exceeding the electrochemical window (Fig. 12c) [73]. An electrochemical reaction, such as charge transfer cross the interface, occurs when the voltage across the EDL exceeds its electrochemical window (~ 1 V) [59]. To ensure electrochemical stability, a common approach to avoid electrochemical reactions has involved designing an electrical circuit to dissipate the voltage applied to the EDL. The relatively high capacitance of the EDL (0.1 F m^−2^) suppresses the electrochemical reaction by maintaining a low voltage drop below 1 V across the EDL. It prevents charge transfer at the interface, thus enabling ionic materials to be utilized even in high-voltage applications. However, this approach is limited by the fact that it is only applicable to certain applications containing a capacitive part connected in series to the EDL.

To reduce electrochemical reaction risks, various studies have been conducted to expand the electrochemical window using organogels or ionogels. These studies demonstrate higher electrochemical stability compared to hydrogels, leading to enhanced system reliability. By using ionic liquids (ILs), for instance, the electrochemical window was increased up to 6 V, ensuring enhanced electrochemical stability [201]. In addition, the systemization of these ionic devices has the potential to reduce electrochemical reactions at the interfaces of electron-based devices. In the future, ionic components such as ionic diodes, actuators, power sources, communicators, computational circuits could be integrated into fully ionic systems [167, 202–207]. Future research focusing on the systemization of ionic devices will provide innovative solutions to address electrochemical reactions at ionic-electronic interfaces.
